# ETS-NOCV description of σ-hole bonding

**DOI:** 10.1007/s00894-012-1591-0

**Published:** 2012-09-30

**Authors:** Karol Dyduch, Mariusz P. Mitoraj, Artur Michalak

**Affiliations:** Department of Theoretical Chemistry, Faculty of Chemistry, Jagiellonian University, R. Ingardena 3, 30-060 Krakow, Poland

**Keywords:** ETS-NOCV, Halogen bonding, Sigma hole bonding

## Abstract

The ETS-NOCV analysis was applied to describe the σ-hole in a systematic way in a series of halogen compounds, CF_3_-X (*X* = I, Br, Cl, F), CH_3_I, and C(CH3)_n_H_3-n_-I (*n* = 1,2,3), as well as for the example germanium-based systems. GeXH_3_, *X* = F, Cl, H. Further, the ETS-NOCV analysis was used to characterize bonding with ammonia for these systems. The results show that the dominating contribution to the deformation density, Δ*ρ*_*1*_, exhibits the negative-value area with a minimum, corresponding to σ-hole. The “size” (spatial extension of negative value) and “depth” (minium value) of the σ-hole varies for different X in CF_3_-X, and is influenced by the carbon substituents (fluorine atoms, hydrogen atoms, methyl groups). The size and depth of σ-hole decreases in the order: I, Br, Cl, F in CF_3_-X. In CH_3_-I and C(CH3)_n_H_3-n_-I, compared to CF_3_-I, introduction of hydrogen atoms and their subsequent replacements by methyl groups lead to the systematic decrease in the σ-hole size and depth. The ETS-NOCV σ-hole picture is consistent with the existence the positive MEP area at the extension of σ-hole generating bond. Finally, the NOCV deformation density contours as well as by the ETS orbital-interaction energy indicate that the σ-hole-based bond with ammonia contains a degree of covalent contribution. In all analyzed systems, it was found that the electrostatic energy is approximately two times larger than the orbital-interaction term, confirming the indisputable role of the electrostatic stabilization in halogen bonding and σ-hole bonding.

**Figure:**
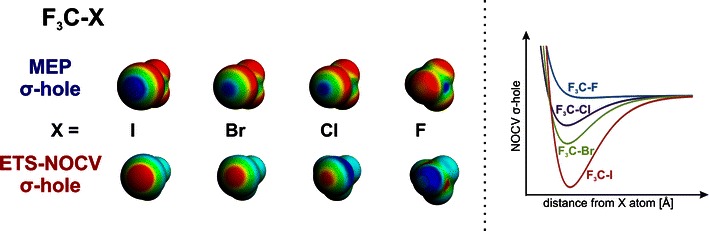
Graphical representation of the σ-hole on the halogen atom, based on the molecular electrostatic potential (upper row) and the NOCV deformation-density channel *Δρ*_*1*_ (lower row and the right-hand side plot)

Graphical representation of the σ-hole on the halogen atom, based on the molecular electrostatic potential (upper row) and the NOCV deformation-density channel *Δρ*_*1*_ (lower row and the right-hand side plot)

## Introduction

Halogen bonds formed between terminal halogen atoms (X) and Lewis bases (B), R–X^**…**^B, play essential role in chemistry and biochemistry [1–8]. In parallel to hydrogen bonding [9, 10], these type of connections attracted recently considerable attention. It has been shown that selective and directional character of these interactions make them very important not only in determination of biological structures but also in rational design of solid materials [1–8].

The first steps toward understanding the geometry and reactivity of halogen bonded systems originate from the works of Hassel et al. [11, 12], Parthasarathy et al. [13], and Mertrangolo et al. [14, 15]. In addition, it is necessary to reference the computational studies of Price et al. [16] and Allen et al. [17]. These works allowed to observe that R–X^**…**^B unit adopts linear structure (the angle R–X^**…**^B is 180°), whereas the electrophilic species (Lewis acids–A) make the R–X^**…**^A angle in the range from 90 up to 120°. In addition the possible role of the electrostatic and the charge transfer components were discussed [16, 17].

The novel concept that explains the origin of halogen bonding was proposed recently by Politzer and coworkers [1, 3, 6, 7, 18–32]. The authors noticed for the first time, based on the molecular electrostatic potential (MEP), anisotropy in distribution of the charge on halogen atom, resulting in the existence of the positive-MEP area on the outer part of halogen atom which is collinear with the R-X bond [1, 6, 7]. Later this phenomenon was connected with an electron deficiency at the outer part of the halogen atom, the so called *σ-hole* [18]. Hence, the halogen atom of R–X is characterized by strong anisotropy of the electron density distribution. The existence of *σ-hole* at terminal halogen atom leads to electrostatic attraction with Lewis bases. Accordingly, the halogen bonding is driven mainly by the electrostatic term [1, 3, 6, 7, 18–32]. Very recently, Politzer and coworkers extended the σ-hole concept to analysis of π-holes [33]. It is noteworthy that non-typical halogen bonds of the type RNC^**…**^FCl have been studied recently by Del Bene et al. [34] and subsequently by Politzer et al. [35] Authors noted very high binding energies (~20–30 kcal mol^−1^) and significant stretch of the F–Cl connection due to formation of RNC^**…**^FCl bond (by ca. 0.3 Å). Politzer and Murray [34] reported, based on the analysis of molecular electrostatic potential and the averaged ionization energies, that it is due to polarization of RNC unit that can lead to significant dative component of RNC^**…**^FCl bond.

We have recently proposed the *natural orbitals for chemical valence* (NOCV) [36, 37] combined with the extended-transition-state (ETS) energy-decomposition analysis [38]. This scheme allows for separation and quantitative assessment of the contributions to deformation density (Δρ_i_) originating from the electron charge transfer channels (σ, π, δ, etc.) [36, 37]. This picture is further enriched by providing the energetic contributions (Δ*E*_*i*_) to the bond energy within ETS-NOCV scheme [39]. It has been shown that NOCV’s lead to a compact description of not only metal–ligand or covalent connections [36, 37, 39], but also of hydrogen bonding [40]. We have verified recently the applicability of ETS-NOCV scheme in a description of halogen bonding, showing in particular, that the dominating contribution to the deformation density exhibits the negative-value area that corresponds to σ-hole [41].

The main goal of the present study is to analyze the ETS-NOCV representation of σ-hole in a more detailed, systematic way in a series of halogen compounds, CF_3_-X (*X* = I, Br, Cl, F), CH_3_I, C(CH3)_n_H_3-n_-I (*n* = 1,2,3), as well as for the example germanium-based systems. XGeH3, *X* = F, Cl, H. The ETS-NOCV scheme will be further used to characterize bonding with ammonia for all analyzed systems.

## Computational details

All the DFT calculations presented here are based on the Amsterdam density functional (ADF2009) program in which ETS-NOCV scheme was implemented [42–45]. The Becke-Perdew exchange-correlation functional [46, 47] was applied (BP86) with an inclusion of the dispersion correction (BP86-D) [48]. A triple-zeta STO basis containing two sets of polarization functions, based on the frozen core approximation, was adopted for all of the elements (TZ2P). Auxiliary *s, p, d, f* and *g* STO functions, centered on all nuclei, were used to fit electron density and obtain accurate Coulomb potentials in each SCF cycle. Relativistic effects were included using the ZORA formalism.

**Fig. 1:**
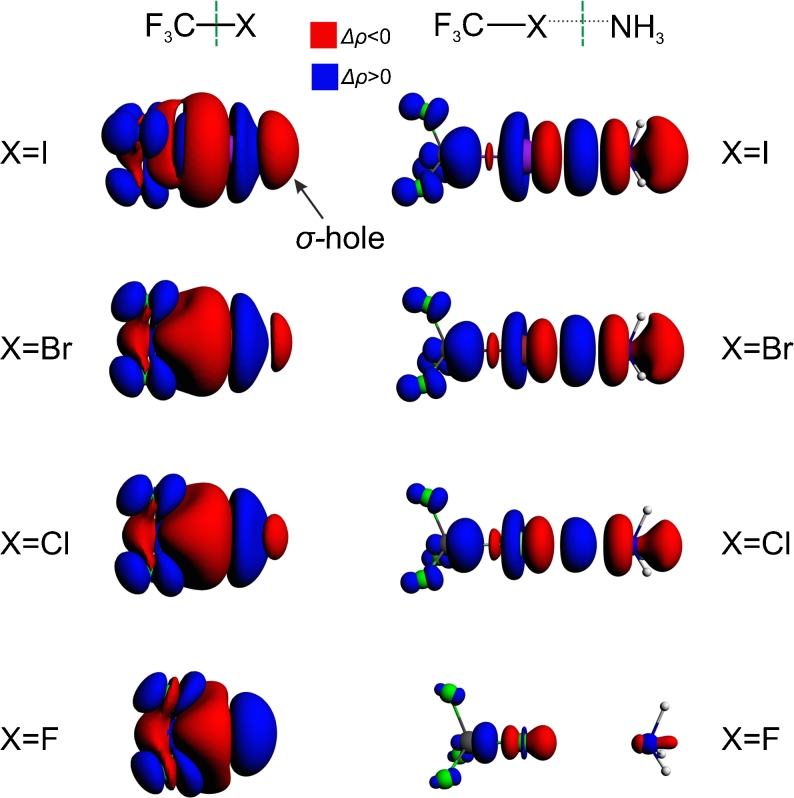
The contours of deformation density contribution *Δρ*_*1*_ characterizing the F_3_C-X bond (left column; the contour values: ±0.0003 a.u.); and the halogen bonding with ammonia in F_3_C-X---NH_3_ (right column; contour values: ±0.0005 a. u.)

Bonding analysis presented in this study is based on the ETS-NOCV approach [39] which is a combination of the extended transition state (ETS) [38] method with the natural orbitals for chemical valence (NOCV) scheme [36, 37]. In our analysis, each system is divided up into two individual fragments as shown schematically by green vertical lines in Fig. 1. Then we used the ETS-NOCV method to study the interaction between these subsystems.

In the ETS energy decomposition scheme [38], the interaction energy Δ*E*_int_ between the fragments (exhibiting geometries as in the combined molecule) is divided into three components:

1 $$ \varDelta {E_{{\mathrm{int}}}} = \varDelta {E_{{\mathrm{elstat}}}} + \varDelta {E_{{\mathrm{Pauli}}}} + \varDelta {E_{{\mathrm{orb}}}}. $$

The first term, Δ*E*_elstat_, corresponds to the classical electrostatic interaction between the fragments as they are brought to their positions in the final molecule. The second term, Δ*E*_Pauli_, accounts for the repulsive Pauli interaction between occupied orbitals on the fragments in the combined molecule. The third stabilizing term, Δ*E*_*orb*,_ represents the interactions between the occupied molecular orbitals of one fragment with the unoccupied molecular orbitals of the other fragments as well as mixing of occupied and virtual orbitals within the same fragment (inner-fragment polarization). This energy term may be linked to the electronic bonding effect coming from the formation of a chemical bond.

The natural orbitals for chemical valence allow for a decomposition of the differential density Δ*ρ* into NOCV-contributions (Δ*ρ*_*k*_):

2 $$ \varDelta \rho (r) = \sum\limits_k^{{NOCV}} {\varDelta {\rho_k}(r)}, $$

based on diagonalization of the deformation-density matrix in the basis set representation [36, 37]. In addition, within the combined ETS-NOCV [39] scheme, the deformation-density based picture is enriched by the energetic estimation, $\varDelta E_{{orb}}^k$,that arises from a decomposition of the aforementioned ETS orbital-interaction energy into the contributions corresponding to NOCV charge-transfer channels, Δ*ρ*_*k*_; $\varDelta E_{{orb}}^k$:

3 $$ \varDelta {E_{{orb}}} = \sum\limits_k {\varDelta E_{{orb}}^k}. $$

It is necessary to mention at this point that the total orbital interaction term includes the inter-fragments electron flow as well as the intra-fragment polarization; thus, depending on the terminology used it could be considered as the polarization energy component [3, 49, 50].

The contours and the color-coded plots of the NOCV deformation density contributions and molecular electrostatic potential were plotted based on ADF-GUI interface [51].

As has already been mentioned, we will use the dispersion corrected BP86-D functional [48], hence, the dispersion correction (Δ*E*_disp_) will be added to Δ*E*_int_ values to describe the total bonding energy:

4 $$ \varDelta {E_{{\mathrm{tot}}}} = \varDelta {E_{{\mathrm{int}}}} + \varDelta {E_{{\mathrm{disp}}}}. $$

## Results and discussion

### ETS-NOCV description of σ-hole in F_3_C-I and the halogen bond in F_3_C-I^…..^NH_3_

Let us start the discussion with two illustrative examples of the ETS-NOCV interpretation of bonding. We will first characterize the bond between the CF_3_ fragment and the iodine atom in F_3_C-I, and then we will describe the interaction between the CF_3_I and ammonia molecule in F_3_C-I^…..^NH_3_. The former example allows us to discuss and visualize the σ-hole in this system, while the latter demonstrates the NOCV picture of halogen bonding [41].

In Fig. 1 (top row, left-hand side plot) we present the contour plot of the dominating NOCV contribution Δ*ρ*_1_ in the deformation density, $\varDelta \rho = {\rho^{{\mathrm{CF3I}}}}--({\rho^{{\mathrm{CF3}}}} + {\rho^{\mathrm{I}}})$. The presented contour shows a formation of covalent C–I σ-bond; the corresponding ETS-NOCV orbital interaction energy is $\varDelta E_{{orb}}^1 = - 98.4kcal/mol$. It should further be noted that the contour of Δ*ρ*_1_ shows significant charge anisotropy around the iodine atom, that is important for the reactivity, as the halogen atom can simultaneously act as electron donor and acceptor [18, 19, 52]. However, a feature of the contour of Δ*ρ*_1_ that is the most important for our further discussion, is an outflow of the electron density from the outer area of iodine atom, at the extension of the C-I bond. Such a negative part of Δ*ρ*_1_ describes the formation of σ-hole within the ETS-NOCV picture.

**Fig. 2:**
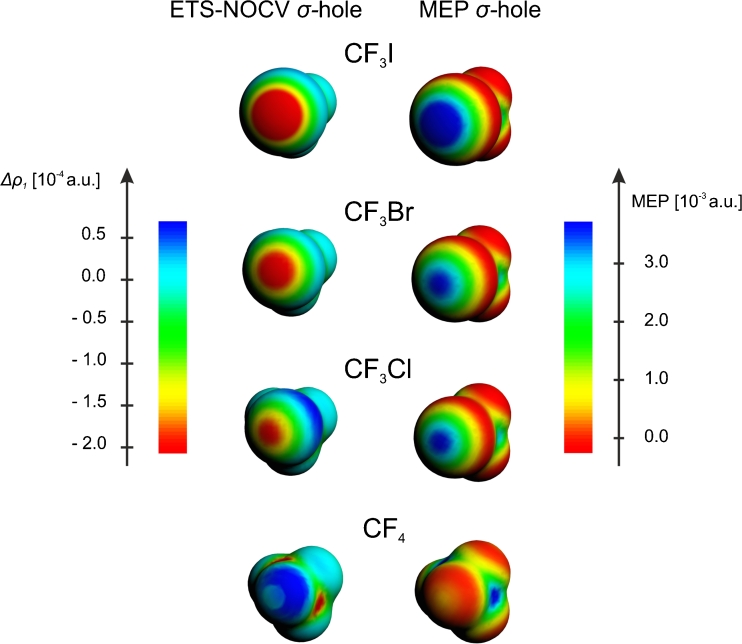
A comparison of the ETS-NOCV deformation-density contributions *Δρ*_*1*_ (*left column*) and the molecular electrostatic potential (right column), presented as color-coded plot on the molecular surface (*ρ* = 0.001 a.u.), for the F_3_C-X systems

In Fig. 2 (top row, left-hand side plot) another graphical representation of the ETS-NOCV σ-hole in CF_3_I is shown: a color-representation of the Δ*ρ*_1_ on the molecular surface (*ρ* = 0.001 a.u.). This plot is compared with the corresponding visualization of molecular electrostatic potential (top row, right-hand side plot). A comparison of the ETS-NOCV and MEP plots demonstrates a clear correspondence of the negative part of Δ*ρ*_1_ with the positive MEP at the tip of the iodine atom.

**Fig. 3:**
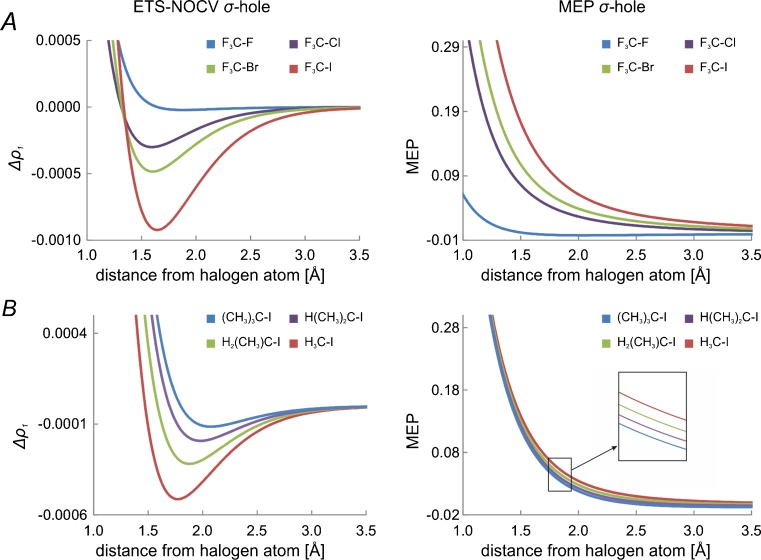
Comparison of the changes in the ETS-NOCV deformation-density contribution *Δρ*_*1*_ (left column) and the molecular electrostatic potential (right column) at the extension of the carbon-halogen bond

Since both, contour plots of Fig. 1 and colored-surface plot of Fig. 2 are quite qualitative, in Fig. 3 we present yet another graphical representation of σ-hole, i.e., the values of Δ*ρ*_1_ along the bond-line, outside of the halogen atom. Here, we can clearly see that Δ*ρ*_1_ exhibits a minimum value, corresponding to σ-hole. Again, in the right-hand side column in Fig. 3, the corresponding MEP plot, as a function of the distance from the iodine atom, is shown. The corresponding numerical values that characterize the Δ*ρ*_1_ minimum, and the MEP value at the point corresponding to the σ-hole minimum are listed in Table 1.

**Table 1:** ETS-NOCV and MEP characteristics of the σ-hole localized on halogen atom in the studied systems

Molecule	*σ*-hole (Δ*ρ*_*1*_) minimum [10^-3^ a.u.]	Minimum distance [Å]	MEP at minimum distance [10^-2^ a.u.]	Halogen-bond energy [kcal/mol]	Halogen bond distance [Å]
CF_3_I	−0.92	1.64	12.50	−6.96	2.862
CF_3_Br	−0.48	1.62	8.15	−4.34	2.857
CF_3_Cl	−0.30	1.60	5.96	−2.59	3.016
CF_4_	−0.02	1.88	−0.22	----	----
C H_3_I	−0.51	1.79	5.27	−3.01	3.086
C(CH_3_)H_2_I	−0.32	1.86	4.25	−2.45	3.175
C(CH_3_)_2_HI	−0.19	1.99	2.26	−2.07	3.174
C(CH_3_)_3_I	−0.11	2.09	1.18	−1.87	3.186

Let us now discuss the ETS-NOCV results for the halogen bond between the CF_3_I and ammonia molecule in F_3_C-I^…..^NH_3_. In Table 2 we collect results of the energy decomposition analysis. Before discussing the results it should be pointed out that in the present study the ZORA relativistic approach was applied; therefore, the presented F_3_C-I^…^NH_3_ bond-energy and its components are slightly different from those of our previous work (not including relativistic correction) [41].

**Table 2:** ETS-NOCV bond-energy decomposition for halogen bonding with ammonia for the set of studied molecules. Values given in kcal mol^–1^. ΔE_orb_^1^ describes the contribution to the orbital interaction energy coming from the dominating NOCV pair

Molecule	Δ*E*_*tot*_	Δ*E*_*disp*_	Δ*E*_*elstat*_	Δ*E*_*Pauli*_	Δ*E*_*orb*_	Δ*E*_*orb*_^*1*^
F_3_C-I^…^NH_3_	−6.96	−1.32	−16.65	20.60	−9.59	−8.90
F_3_C-Br^…^NH_3_	−4.34	−1.02	−10.44	12.90	−5.78	−5.48
F_3_C-Cl^…^NH_3_	−2.59	−0.81	−5.03	5.75	−2.51	−2.43
F_3_C-F^…^NH_3_	*(Repulsive interaction energy; no halogen-bond minimum found)*					
H_3_C-I^…^NH_3_	−3.01	−1.19	−8.05	10.72	−4.49	−3.95
H_2_(CH_3_)C-I^…^NH_3_	−2.46	−1.21	−5.93	8.18	−3.50	−3.14
H(CH_3_)_2_C-I^…^NH_3_	−2.07	−1.24	−5.90	8.65	−3.57	−3.12
(CH_3_)_3_C-I^…^NH_3_	−1.87	−1.25	−5.60	8.49	−3.51	−3.09

As shown in Table 1, the total interaction energy between F_3_C-I and NH_3_ is −7.0 kcal mol^−1^. The results show that the total stabilization of the system is mainly due to the electrostatic interaction (-16.7 kcal mol^−1^), that is almost two times larger than the orbital interaction energy (−9.6 kcal mol^−1^), in agreement with the previous studies emphasizing the role of electrostatic stabilization [18, 19, 41].

The dispersion energy, −1.3 kcal mol^−1^, is of minor importance here, as it was shown in the case of other systems with σ-hole bonding [18, 19, 41, 53]. It should, however, be noted that Riley et al. [53] studied similar molecules (CY_3_X----OCH_2_, *X* = Cl, Br, I, *Y* = H, F) containing formaldehyde donor, based on SAPT-energy decomposition scheme – for these molecules both the electrostatic and the dispersion factors were found to be similarly important (moreover, for *X* = Cl and *Y* = H,F, the dispersion was visibly more important than the electrostatic term) [53]. It is also necessary to note that due to inherent methodological differences between SAPT and DFT-D approaches one can obtain different absolute values characterizing the role of dispersion [54]. Role of the dispersion in the aromatic compounds was recently characterized by Riley et al. [55].

The leading NOCV deformation density channel, Δ*ρ*_1_, that describes the halogen bonding between CF_3_I molecule and ammonia, is shown in Fig. 1 (top row, right-hand side plot). The contour plot of Δ*ρ*_1_ shows the *covalent bonding* contribution that originates from the electron transfer from both the nitrogen and iodine atoms to the bonding region between I and N atoms. Also, the *donation* from the NH_3_ fragment to CF_3_I is clearly visible, with the decrease in the electron density on ammonia and the increase in the region of the CF_3_ group. Accumulation of electron density at carbon atom of CF_3_ is in line with an increase in the carbon *s*–orbital contribution due to halogen bonding, shown recently by Grabowski with NBO method [52].

It should be emphasized that Δ*ρ*_1_ is the only NOCV contribution localized in the halogen bond area, with the corresponding energy contribution *ΔE* ^*1*^_*orb*_ *= −*8.9 kcal mol^−1^, that covers over 93 % of the orbital interaction energy; the remaining NOCV channels (not shown) describe the intra-fragment polarization and are characterized by much lower energies (stabilization up to −0.2 kcal mol^−1^).

### Comparison of σ-hole in F_3_C-X and halogen bonding with NH_3_, for *X* = I, Br, Cl, F

Similarly to CF_3_I molecule, we used ETS-NOCV method to characterize σ-hole and halogen bonding in the analogous systems involving other halogen atoms, *X* = Br, Cl, F in CF_3_X. The contours of the main NOCV charge-transfer channel (Δ*ρ*_1_) for all the systems are collected in the left-hand side column of Fig. 1. The corresponding color-coded representation of Δ*ρ*_1_ is presented Fig. 2 (left-hand side column), together with the similar MEP plots (right-hand side column). In panel **a** of Fig. 3, the linear Δ*ρ*_1_ plots at the extension of the C-X bond (as a function of the distance from atom X) are compared for the systems with different halogens. The corresponding numerical values that characterize the Δ*ρ*_1_ minimum, and the MEP value at the point corresponding to the σ-hole minimum are listed in Table 1.

The results of Figs. 1, 2, 3a, and Table 1 show consistently a decrease in the σ-hole “depth” (Δ*ρ*_1_ minimum value) and “size” (spatial extension of the negative Δ*ρ*_1_ area) in the order: I, Br, Cl. In particular, the decrease of the σ-hole size is clear from the contour plot of Fig. 1 and the color-coded plot of Fig. 2, while the decrease in σ-hole depth is nicely demonstrated by the linear plots of Fig. 3a. The minimum values of Δ*ρ*_1_ are −0.92*10^−3^ a.u.; −0.48*10^−3^ a.u., and −0.30*10^−3^ a.u., for I, Br, and Cl, respectively (see Table 1). When going from I to Cl, the position of the minimum moves closer to the halogen atom; the position, measured as the distance from the atom X, changes from 1.64 Å for I, through 1.62 Å for Br, to 1.60 Å for Cl. For fluorine, in the case of the contour of Δ*ρ*_1_ (Fig. 1) and its color-coded representation (Fig. 2), practically no σ-hole (negative Δ*ρ*_1_) is visible; a very shallow minimum of Δ*ρ*_1_ (−0.02*10^-3^ a.u.) appears at a relatively large distance of 1.88 Å.

The picture resulting from ETS-NOCV analysis concerning σ-hole in the CF_3_X systems are fully consistent with the results of the previous studies based on analysis of MEP [1] and NBO [18]. To compare the NOCV and MEP picture in a more detailed way, based on the calculations within the same methodology, in the right-hand columns of Figs. 2 and 3 the corresponding MEP plots are presented; the numerical values of MEP at the point corresponding to the minimum of Δ*ρ*_1_ are in addition listed in the last column of Table 1. A comparison of Δ*ρ*_1_ and MEP plots in Fig. 2 show a nice qualitative correspondence of the negative Δ*ρ*_1_ and positive MEP areas at the tip of the halogen atom. The plots of Fig. 3 and the values from Table 1 show a decrease of MEP values in the order I > Br > Cl > F; the MEP values at the Δ*ρ*_1_ mininum changes from 0.125 a.u. for I, through 0.082 a.u. for Br, down to 0.060 a.u. for Cl. For the system with fluorine, the MEP is no longer positive at the tip of the halogen atom, but becomes negative; the MEP value at the minimum of Δ*ρ*_1_ is −0.002 a.u.

The trends in σ-hole, discussed above, are reflected by the halogen bond energies in the F_3_C-X interacting with ammonia. The stabilization in the total bond energy decreases in the order: F_3_C-I (−7.0 kcal mol^−1^), F_3_C-Br (−4.3 kcal mol^−1^), F_3_C-Cl (−2.6 kcal mol^−1^). For F_3_C-F---NH_3_ there is practically no bonding; no minimum corresponding to the halogen bond was found within the used DFT approach. We will comment on the F_3_C-F---NH_3_ interaction in a more detail later.

As for the iodine-system discussed in the previous section, for X = Br and Cl the electrostatic contribution is roughly two times larger (−10.4, −5.0 kcal mol^−1^ for Br, Cl, respectively) than the orbital interaction term (−5.8, −2.5 kcal mol^−1^ for Br, Cl, respectively). The most important NOCV charge-transfer channel, Δ*ρ*_1_, accounts for 93 %, 95 %, and 97 % of the orbital interaction contribution, for I, Br, and Cl (*ΔE* ^*1*^_*orb*_ *= −*8.9, −5.5, and −2.4 kcal mol^−1^, respectively). The NOCV plots in the left-hand side column of Fig. 1 clearly show that the NOCV picture of halogen bonding is qualitatively the same for I, Br, and Cl: for all those systems the covalent component of halogen bond is seen; only the spatial extension of the corresponding Δ*ρ*_1_ contours decreases.

Let us now discuss the F_3_C-F---NH_3_ system. As we mentioned above, the DFT methodology applied here does not allow to find a minimum corresponding to the halogen bonding. In addition to the standard, unconstrained geometry optimization, we have performed a set of constrained optimizations, with a frozen, increasing F-N distance, starting from the linear C-N-F structure with *R*_F-N_ = 2.00 Å. Up to the distance of 2.24 Å the systems stays roughly linear, and afterward the C-N-F angle starts strongly deviate from linearity. The interaction between CF_4_ and ammonia is repulsive within used methodology (e.g., at *R*_F-N_ = 2.1 and 2.2 Å the total bonding energy Δ*E*_*tot*_ is *+*0.2 and +0.1 kcal mol^−1^, respectively); the dispersion energy is slightly stabilizing (−0.5 kcal mol^−1^ and −0.4 kcal mol^−1^, at 2.1 Å and 2.2 Å, respectively), but not large enough to overcome the repulsive part of the interaction energy. Certainly, these small energy values are not quantitatively meaningful, and they will be strongly influenced by the methodology used, so that we do not discuss all the interaction-energy components here. Our point is just to qualitatively illustrate the lack of pronounced halogen bonding for F_3_C-F---NH_3_. Further, in the last column of Fig. 1 (right-hand side plot), for comparison with other systems, we plotted the example of the main NOCV Δ*ρ*_1_ channel for *R*_F-N_ = 2.15 Å, that shows only the intra-fragment polarization, without the covalent halogen-bonding features; it should be emphasized that for other points that we analyzed (for *R*_F-N_ = 2.00 − 2.24 Å) the corresponding Δ*ρ*_1_ plots are qualitatively indistinguishable.

Therefore, it may be concluded that in CF_3_-X systems the ETS-NOCV analysis shows, in agreement with previous studies [1, 18], that the size and depth of σ-hole decreases in the order X = I, Br, Cl, F. This trend is reflected by the changes in MEP, as well as the changes in the NOCV Δ*ρ*_1_ component in F_3_C-X---NH_3_, and the strength of the interaction with ammonia.

### Influence of carbon-substituents in R_1_R_2_R_3_C-I on σ-hole and halogen bonding

**Fig. 4:**
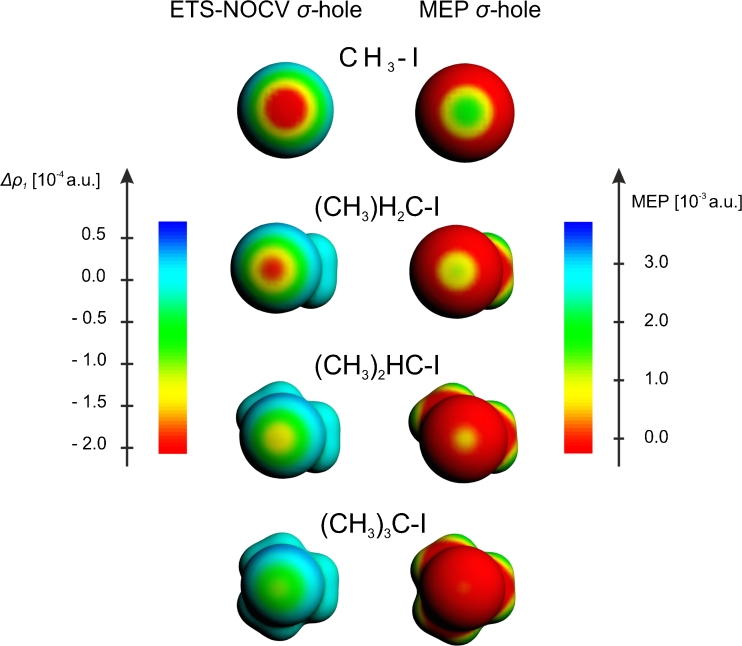
A comparison of the ETS-NOCV deformation-density contributions *Δρ*_*1*_ (*left column*) and the molecular electrostatic potential (*right column*), presented as color-coded plot on the molecular surface (*ρ* = 0.001 a.u.), for the (CH_3_)_x_H_3-x_C-I systems

After discussing the σ-hole and halogen bonding in CF_3_X systems, now we would like to present the ETS-NOCV results for the related CH_3_I, and C(CH3)_n_H_3-n_-I (*n* = 1,2,3) molecules, in which the fluorine atoms of CF_3_I are first replaced by hydrogen atoms, and then further by methyl groups. The respective plots describing ETS-NOCV σ-hole are shown and compared with the corresponding MEP plots in Figs. 3b and 4; the respective numerical data are collected in Tables 1 and 2.

A comparison of the results for CF_3_I (Fig. 2, 3a, Table 1) and CH_3_I (Fig. 3b, 4, Table 1) show that the exchange of fluorine atoms with hydrogens has a relatively strong effect on the iodine σ-hole: the minimum changes from −0.9*10^−3^ a.u. in CF_3_I to -0.5*10^−3^ a.u. CH_3_I. This is consistent with MEP picture: the value of MEP at the Δ*ρ*_1_ minimum decreases from 0.125 a.u. in CF_3_I to 0.053 CH_3_I.

Consistently, a similar effect is observed for the halogen bond energies and their components (Table 2). The total stabilization due to halogen bonding decreases by ca. 4.0 kcal mol^−1^ (from −7.0 kcal mol^-1^ to −3.0 kcal mol^−1^ in CH_3_I). The corresponding decrease in stabilization due to orbital interaction is 5.1 kcal mol^-1^ (from the value of −9.6 kcal mol^−1^ in CF_3_I to −4.5 kcal mol^−1^ in CH_3_I). The decrease in the electrostatic stabilization is 8.5 kcal mol^−1^ (from the value of −16.6 kcal mol^−1^ in CF_3_I to −8.1 kcal mol^−1^ in CH_3_I). Thus, the ETS-NOCV analysis provides similar conclusions to those previously published by Politzer et al. [1].

Introducing the methyl substituents on carbon leads to the further decrease in the σ-hole depth. For subsequent methyl substitution the minimum value of Δ*ρ*_1_ changes in the sequence: -0.3*10^−3^ a.u., −0.2*10^−3^ a.u., −0.1*10^−3^ a.u. in C(CH_3_)H_2_I, C(CH_3_)_2_HI, and C(CH_3_)_3_I, respectively. Again, the data of Tables 1 and 2 show that this is reflected by the changes in MEP at the σ-hole minimum (0.042, 0.022, 0.012 a.u.. respectively), as well as by the halogen-bond energies (−2.5, −2.0, -1.9 kcal mol^−1^, respectively) and their components.

### σ-hole in GeH_3_-X and bonding with NH_3_

Although the concept of σ-hole was proposed originally to rationalize the halogen bonding, it was shown later by Politzer and coworkers [3, 22, 33, 34] that it is useful as well for explanation of the weak, bonding interactions in other systems. Therefore, we would like here to use ETS-NOCV to describe and visualize σ-hole in the example germanium compounds, GeH_3_X, X = F, Cl, H, and their interaction (σ-hole bonding) with ammonia molecule. It was shown recently [22] that the molecular electrostatic potential exhibits the positive-value area on the outer part of germanium atom, at the extension of the X-Ge bond (i.e., between three Ge-H bonds); this was rationalized by the σ-hole concept, analogous to those observed at halogen atom in the compounds that can form halogen bonding [18–21].

**Fig. 5:**
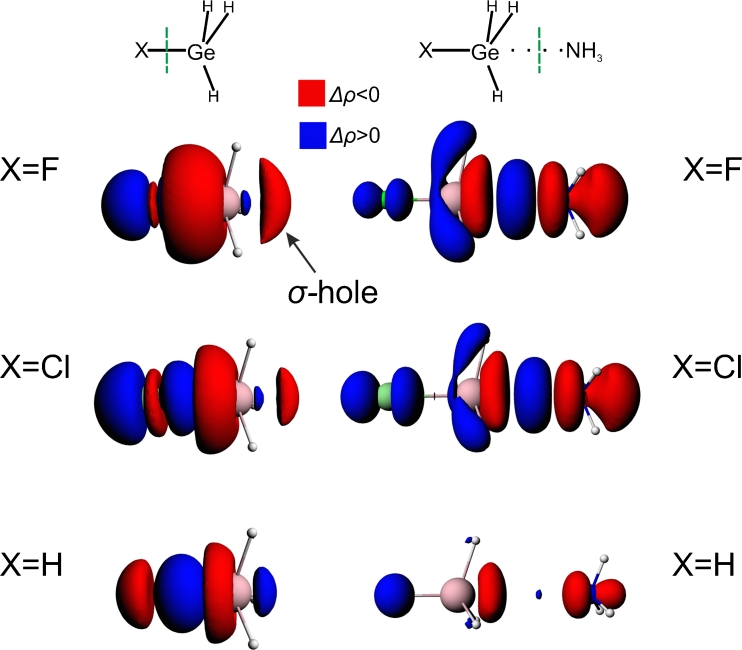
The contours of deformation density contribution *Δρ*_*1*_ characterizing the X-GeH_3_ bond (left column; the contour values: ±0.002 a.u.); and the σ-hole-bonding with ammonia in XH_3_Ge---NH_3_ (right column; contour values: ±0.0005 a. u.)

**Fig. 6:**
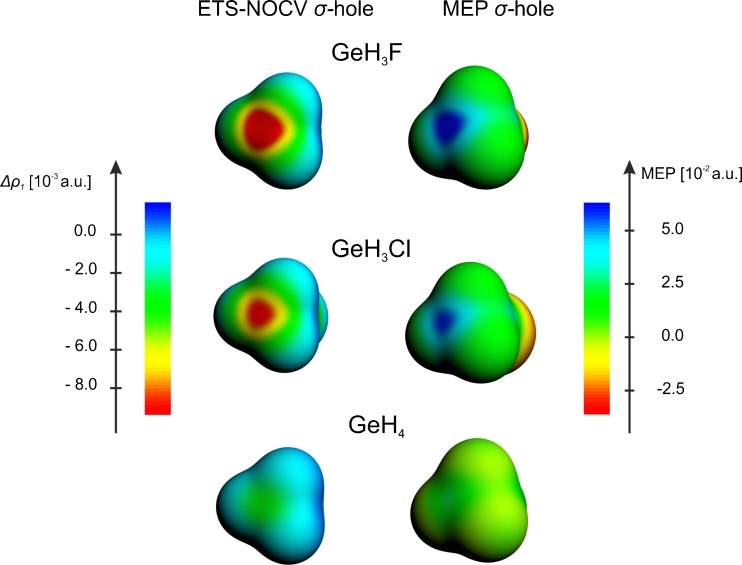
A comparison of the ETS-NOCV deformation-density contributions *Δρ*_*1*_ (*left column*) and the molecular electrostatic potential (*right column*), presented as color-coded plot on the molecular surface (*ρ* = 0.001 a.u.), for the germanium-based systems

**Fig. 7:**
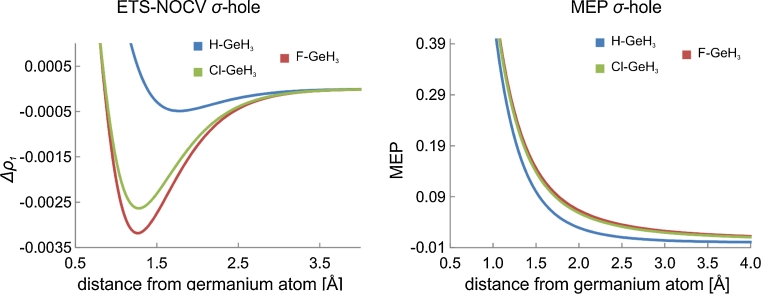
Comparison of the changes in the ETS-NOCV deformation-density contribution *Δρ*_*1*_ (left column) and the molecular electrostatic potential (right column) at the extension of the X-Ge bond (*X* = F,Cl,H)

In order to describe σ-hole formation within the ETS-NOCV scheme, we again apply the two fragment approach, with X and GeH_3_ considered as the fragments. Similarly to the systems described in the previous sections, we present in the left-hand side column of Fig. 5 the contours of the dominant NOCV deformation-density contribution of σ-symmetry, Δ*ρ*_1_, for the X-GeH_3_. The color-coded representation of Δ*ρ*_1_ is shown in Fig. 6, and compared with the corresponding MEP plots. Finally, in Fig. 7, the linear plots of Δ*ρ*_1_ as a function of the distance from germanium atom are presented and compared with the corresponding MEP curves. The numerical values characterizing the NOCV σ-hole minimum and MEP are listed in Table 3. The σ-hole bonding with ammonia is described by the contours of Δ*ρ*_1_ for XH_3_Ge---NH_3_ (right-hand column of Fig. 5), and the ETS-NOCV bond-energy components are listed in Table 4.

**Table 3:** ETS-NOCV and MEP characteristics of the σ-hole localized on the germanium atom (on the extension of X-Ge bond), and the σ-bonding with ammonia, for the analyzed X-H_3_Ge systems

Molecule	*σ*-hole (Δ*ρ*_*1*_) minimum [10^-3^ a.u.]	Minimum distance [Å]	MEP at minimum distance [10^-2^ a.u.]	σ-hole-bond energy [kcal/mol]	σ-hole-bond distance [Å]
GeH_3_F	−3.18	1.28	24.85	−5.74	2.646
GeH_3_Cl	−2.63	1.25	24.65	−5.32	2.742
GeH_4_	−0.49	1.76	5.07	−1.13	3.261

**Table 4:** ETS-NOCV bond-energy decomposition for the σ-hole-bonding with ammonia for germanium-based systems. Values given in kcal mol^-1^. ΔE_orb_^1^ describes the contribution to the orbital interaction energy coming from the dominating NOCV pair

Molecule	Δ*E*_*tot*_	Δ*E*_*disp*_	Δ*E*_*elstat*_	Δ*E*_*Pauli*_	Δ*E*_*orb*_	Δ*E*_*orb*_^*1*^
FH_3_Ge^…^NH_3_	−5.74	−2.30	−20.32	26.22	−9.34	−8.02
ClH_3_Ge^…^NH_3_	−5.32	−2.14	−16.32	20.80	−7.66	−6.58
H_4_Ge^…^NH_3_	−1.13	−1.60	−3.81	5.85	−1.57	−1.27

The results show that, the main Δ*ρ*_1_ ETS-NOCV channel exhibits for FH_3_Ge the extended negative-value area that corresponds to σ-hole (Fig. 5). In the color-coded representation on the molecular surface (Fig. 6), this area corresponds to the MEP-positive-value area. For ClH_3_Ge the σ-hole size (spatial extension) and depth (minimum value) are smaller than for the system with fluorine (Figs. 5, 6, 7); the Δ*ρ*_1_ minimum value changes from −3.2*10^−3^ a.u. (at 1.28 A) for FH_3_Ge to −2.63*10_−3_ a.u. (at 1.25 A) in ClH_3_Ge. This corresponds to the decrease in MEP at the minimum from 0.248 a.u. to 0.247, respectively. In the case of H_4_Ge a very shallow minimum of Δ*ρ*_1_ (−0.5*10^−3^ a.u.) is observed at a relatively long distance from germanium (1.76 A); it corresponds to a small positive value of MEP (0.051 a.u.).

The presence of σ-hole in XH_3_Ge, X = F, Cl, is responsible for stabilizing interaction with ammonia and correlates qualitatively with the σ-hole-bonding energy (−5.8 kcal mol^−1^ for F and −5.3 kcal mol^−1^ for Cl). The ETS analysis shows that the electrostatic energy is approximately two times larger that the orbital interaction component, similarly to the systems discussed in the previous sections: for X = F the electrostatic contribution is −20.3 kcal mol^−1^ and the orbital-interaction energy is −9.3 kcal mol^−1^; for X = Cl the two components are −16.3 kcal mol^-1^ and −7.7 kcal mol^−1^, respectively. The NOCV Δ*ρ*_1_ contour (right-hand side part of Fig. 5) exhibits features qualitatively similar to the systems described in the previous sections: the covalent Ge-N bond and the charge-transfer H_3_N➔GeH_3_X.

In the case of GeH_4_, the interaction with ammonia is slightly attractive (−1.1 kcal mol^−1^) due to dispersion (−1.6 kcal mol^−1^); the sum of the remaining components is repulsive (+0.5 kcal mol^−1^). A relatively weak electrostatic (−3.8 kcal mol^−1^) and orbital interaction (−1.6 kcal mol^−1^) contributions are not large enough to overcome the Pauli repulsion (5.9 kcal mol^−1^). The Δ*ρ*_1_ contour exhibit a very small covalent-bonding area, being dominated by the intra-fragment polarization.

As for halogen bonding discussed in the previous sections, the results presented here for the ETS-NOCV description of the σ-hole in germanium compounds and their σ-hole-bonding with ammonia are in a qualitative agreement with the picture emerging from the previous studies [1–4, 18–34], emphasizing the role of σ-hole formation for the electrostatic stabilization of bonding.

### Polarization effect on σ-hole and halogen bonding

It has been shown in ref [56] that the electric field can induce the σ-hole. Accordingly, polarization of one fragment by the interacting, partner fragment is an important factor in determining the strength and direction of hydrogen (and by implication halogen) bonds. In a similar manner we will characterize here the influence of polarization on the NOCV-picture of σ-hole in CF_3_I. Further, the ETS-NOCV description using the *mutually-polarized fragments* will be presented for CF_3_I---NH_3_ bonding, and compared with the non-polarized case.

**Fig. 8:**
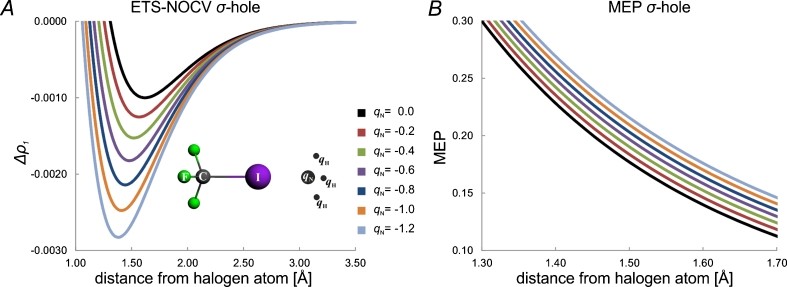
Changes in the ETS-NOCV deformation-density contribution Δρ_1_ (panel A) and the molecular electrostatic potential (panel B) for F_3_C-I at the extension of C–I bond, due to electrostatic field of ammonia, modeled by point charges *q*_*N*_ = −1/3*q*_*H*_; for *q* changing between 0 and 1.2. The black line (*q = 0*) corresponds to non-polarized system (shown in panel A of Fig. 3 – red line)

In order to polarize CF_3_I molecule we used the point-charge model, with point-charges placed in the position of the atoms of NH_3_ : *q*_*N*_ = −*q*; *q*_*N*_ = +*q/3*, for *q* changing between 0.0 and 1.2 . The results are presented in Fig. 8.

As it can be seen from panel A of Fig. 8 the polarization by ammonia increases the “depth” of σ-hole on iodine: the ∆ρ_1_ minimum-value becomes more negative with the increase in the negative charge (on ammonia nitrogen atom). It is fully consistent with the picture emerging from the molecular electrostatic potential presented in panel B of Fig. 8, demonstrating increase of the positive MEP. Thus, the NOCV analysis shows the polarization-induced (or here - enhanced) σ-hole, in a qualitative agreement the data presented in ref [56].

**Fig. 9:**
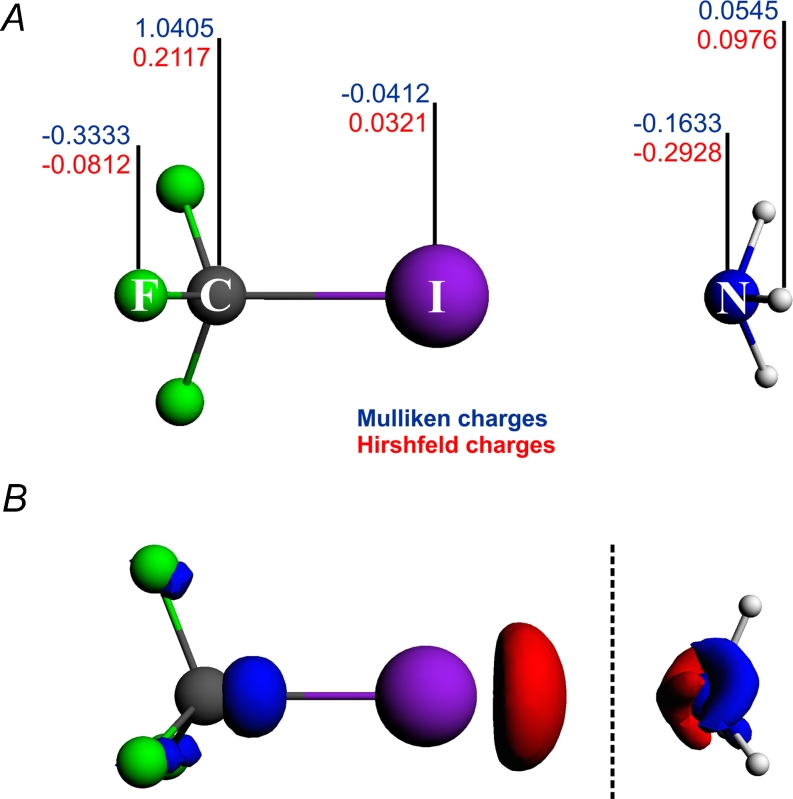
Halogen bonded system, CF_3_I---NH_3_, together with Mulliken (blue color) and Hirshfeld (red color) atomic charges (panel A), used to model the mutual polarization of the fragments. In panel B the contour of deformation density, $\varDelta {\rho_{{pol}}}$, describing the polarization of the CF_3_I and NH_3_ fragments is presented; $\varDelta {\rho_{{pol}}} = ({\rho_{{C{F_3}I - polarized - by - N{H_3}}}} - {\rho_{{C{F_3}I}}}) + ({\rho_{{N{H_3} - polarized - by - C{F_3}I}}} - {\rho_{{N{H_3}}}})$

It is interesting at this point to analyze how the ETS-NOCV description of CF_3_I---NH_3_ bonding will change upon considering the polarized fragments in ETS-NOCV calculations (as compared to non-polarized species NH_3_ and CF_3_I). In order to achieve this goal we have first performed the calculations for the fragments polarized by the point-charges in the positions of the other-fragment atoms: NH_3_ in the field of point charges placed in the atomic positions of CF_3_I; and for CF_3_I fragment in the field of point charges placed in the atomic positions of ammonia. Mulliken and Hirshfeld charges were condidered, as presented in part A of Fig. 9. Subsequently, we applied ETS-NOCV analysis using the polarized-fragments as a reference. The results are presented in Table 5. The fragment density change due to polarization is shown in Fig. 9.

**Table 5:** ETS-NOCV bond-energy decomposition of halogen bonding CF_3_I---NH_3_ when using non-polarized and polarized molecular fragments. Polarization based on Mulliken and Hirshfeld charges were considered. Values given in kcal mol^1^. In paranthesis the diffrence in energy contributions between polarized and non-polarized case is presented

Fragments	Δ*E*_*tot*_	Δ*E*_*disp*_	Δ*E*_*elstat*_	Δ*E*_*Pauli*_	Δ*E*_*orb*_	Δ*E*_*orb*_^*1*^
Non-polarized	−6.96(0.0)	−1.32(0.0)	−16.65(0.0)	20.60(0.0)	−9.59(0.0)	−8.90(0.0)
Mulliken charges	−7.03(−0.07)	−1.32(0.0)	−17.20(−0.55)	20.72(0.12)	−9.23(0.36)	−8.60(0.30)
Hirshfeld charges	−7.12(−0.16)	−1.32(0.0)	−17.33(−0.68)	20.59(−0.01)	−9.05(0.54)	−8.43(0.47)

It can clearly be seen from Table 5 that applying polarized fragments leads to a slightly more stabilizing value of the total intreaction energy as compared to the results based on non-polarized fragments, i.e., Δ*E*_*tot*_ is lower by 0.16 kcal mol^−1^ for Hirshfeld charges and by 0.07 kcal mol^−1^ for Mulliken charges. The increase in the stabilization energy comes from the fact that the reference polarized fragments are higher in energy than non-polarized fragments (optimized KS electron density corresponding to the minimum of energy).

Now, it is interesting to examine the changes in the ETS-bond-energy components due to polarization of the fragments. The results of Table 5 show that the increase in the bond stabilization is almost solely due to an increase in the stabilization originating from the electrostatic contribution (Δ*E*_*elstat*_ is lowered by 0.55 – 0.68 kcal mol^−1^). This result is consistent with the polarization-picture of σ-hole discussed above: the polarization (the partner-electric-field) induced increase in the σ-hole “depth” and the corresponding increase in the positive MEP at the tip of iodine is reflected by the increased electrostatic stabilization of interaction with ammonia (negatively charged nitrogen atom).

Further, the results of Table 5 show that the orbital interaction term (ΔE_orb_) appears to be less stabilizing when going from non-polarized to polarized fragments (by 0.36 kcal mol^−1^ for Mulliken charges and by 0.54 kcal mol^−1^ for Hirshfeld charges). This result may appear surprising and counter-intuitive at the first look: one might expect the increase in orbital-interaction when the σ-hole becomes “deeper”. However, after analyzing this effect in details, it seems to be physical and correct, and in fact could be expected. Namely, when we express the density changes and the bond-energy using *the non-polarized fragments as the reference*, the mutual fragment polarization is included in the NOCV density-changes, and the fragment-polarization energy is included in the orbital-interaction energy term. When, in the second-case, we express the density changes and the bond-energy using *the polarized fragments as the reference*, the mutual fragment polarization (or rather, its part due to the partner-electric-field) is excluded from both, NOCV density changes and the orbital-interaction-energy contribution. As a result, since part of the stabilization energy has already been subtracted from the orbital-intaraction energy by considering polarized fragments, its final value becomes less negative in the polarized-fragment reference case than in the non-polarized fragment case.

The above reasoning is further confirmed when we inspect the polarization changes in the fragment-electron densities, see part B of Fig. 9, it is clearly seen that inclusion of the point charges at positions of neighboring fragments leads to the charge outflow from I–N bonding region, hence, the weakening/destabilization of σ-bonding is observed, ΔE_pol_ = +0.07 kcal mol^−1^ (Mulliken), ΔE_pol_ = +0.16 kcal mol^−1^ (Hirshfeld).

The remaining bonding components (ΔE_disp_ and ΔE_Pauli_) are practically the same. It should finally be mentioned that the polarization not involved in the σ-component of CF_3_I---NH_3_ bonding, measured by difference Δ*E*_*orb*_ *–* Δ*E*_*orb*_^*1*^, also practically does not change when we consider polarized fragments (0.6–0.7 kcal mol^−1^ in both cases).

Summarizing, the above results show that mutual polarization of the NH_3_ and CF_3_I fragments is one of the factors stabilizing halogen bonding, by increase in the σ-hole “depth” and the electrostatic-interaction-energy component. Here, however, we considered only one example; more studies for other systems are required.

## Conclusions

In our previous article [41], we have shown that ETS-NOCV approach can be used to successfully describe halogen bonding, and that the dominating contribution to the deformation density exhibits the negative-value area that corresponds to σ-hole. In the present study we analyzed the ETS-NOCV representation of σ-hole in a more detailed, systematic way in a series of halogen compounds, CF_3_-X (*X* = I, Br, Cl, F), CH_3_I, and C(CH3)_n_H_3-n_-I (*n* = 1,2,3) as well as for the example germanium-based systems. XGeH_3_, *X* = F, Cl, H. We also used ETS-NOCV scheme to characterize bonding with ammonia for these systems. In particular, we have used three different graphical representations of the ETS-NOCV results useful for qualitative and quantitative characterization of σ-hole: the contour plots, color-coded molecular surface, and the linear plots of Δ*ρ*_*1*_ at the extension of the σ-hole-generating bond.

The results of presented analysis showed that the dominating contribution to the deformation density, Δ*ρ*_*1*_, exhibits the negative-value area (σ-hole area) with a minimum. It was shown that the “size” (spatial extension of negative value) and “depth” (minium value) of the σ-hole varies for different X in CF_3_-X, and is influenced by the carbon substituents (fluorine atoms, hydrogen atoms, methyl groups). In particular, the size and depth of σ-hole decreases in the order I, Br, Cl, F in CF_3_-X. In CH_3_-I and C(CH3)_n_H_3-n_-I, compared to CF_3_-I, introduction of hydrogen atoms and their subsequent replacements by methyl groups lead to the systematic decrease in the σ-hole size and depth.

**Fig. 10:**
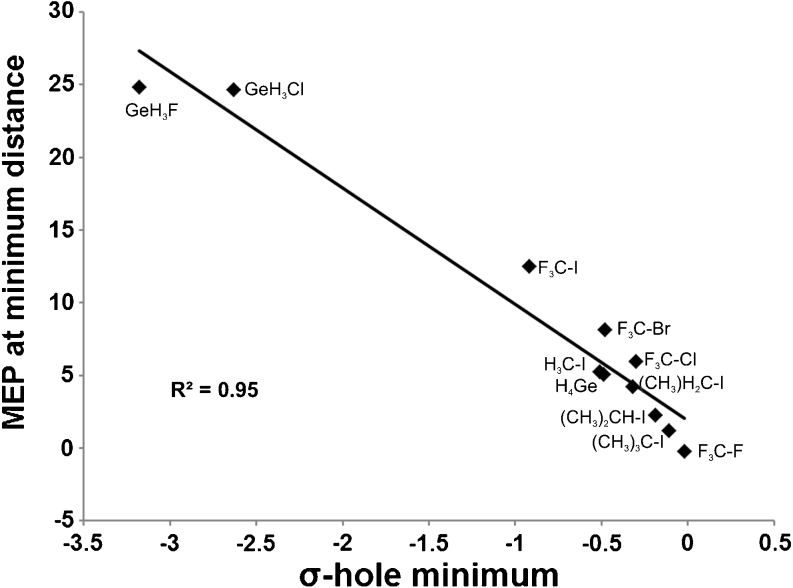
Correlation between the σ-hole minimum (minumim value of the ETS-NOCV deformation-density contribution, *Δρ*_*1*_) and the MEP value at the minimum-point for all studied systems (see Table 1).

It was further shown that the size and depth of the ETS-NOCV representation of σ-hole by Δ*ρ*_*1*_ corresponds qualitatively to the positive MEP area at the extension of σ-hole generating bond, demonstrating, in agreement with the previous works [1, 18], that existence of σ-hole is responsible for this important feature of MEP. To further strengthen this point in Fig. 10 we show the correlation between the minimum value of Δ*ρ*_*1*_ and the MEP value at the Δ*ρ*_*1*_ minimum.

Finally, our results show that the σ-hole bond with ammonia contains a large degree of covalent contribution. It should be emphasized, that any energy partitioning method includes some arbitrariness due to the fact that the contributions to the total interaction energy are not physical observables. However, the presence of the covalent contribution in σ-hole bonding was demonstrated not only by the ETS orbital-interaction energy, but as well by the NOCV deformation density contours. Concerning the interaction-energy components, in all analyzed systems, it was found that the electrostatic energy is approximately two times larger than the orbital-interaction term. Thus, the results of the present analysis confirm the indisputable role of the electrostatic stabilization in halogen bonding and σ-hole bonding, emphasized in the previous articles by Politzer and coworkers [1–4, 18–34].
